# Role of the Gut Microbiota in Children with Kidney Disease

**DOI:** 10.3390/children10020269

**Published:** 2023-01-31

**Authors:** You-Lin Tain, Chien-Ning Hsu

**Affiliations:** 1 Division of Pediatric Nephrology, Kaohsiung Chang Gung Memorial Hospital, Kaohsiung 833, Taiwan; 2 Institute for Translational Research in Biomedicine, Kaohsiung Chang Gung Memorial Hospital, Kaohsiung 833, Taiwan; 3 College of Medicine, Chang Gung University, Taoyuan 333, Taiwan; 4 Department of Pharmacy, Kaohsiung Chang Gung Memorial Hospital, Kaohsiung 833, Taiwan; 5 School of Pharmacy, Kaohsiung Medical University, Kaohsiung 807, Taiwan

**Keywords:** chronic kidney disease, uremic toxin, gut microbiota, short-chain fatty acid, children, trimethylamine N-oxide, tryptophan

## Abstract

Disruption of the composition and structure of the gut microbiota, namely dysbiosis, dictates the pathophysiology of kidney diseases. The bidirectional kidney–gut axis is of interest in chronic kidney disease (CKD); the uremic milieu leads to intestinal dysbiosis and gut microbial metabolites and toxins implicated in the loss of kidney function and increased comorbidity burden. Considering that kidney diseases can originate in childhood or even earlier in fetal life, identification of the pathogenetic connection between gut microbiota dysbiosis and the development of pediatric renal diseases deserves more attention. This review concentrates on the pathogenic link between dysbiotic gut microbiota and pediatric renal diseases, covering CKD, kidney transplantation, hemodialysis and peritoneal dialysis, and idiopathic nephrotic syndrome. Gut microbiota-targeted therapies including dietary intervention, probiotics, prebiotics, postbiotics and fecal microbial transplantation are discussed for their potential for the treatment of pediatric renal diseases. A deeper understanding of gut microbiota in pediatric renal diseases will aid in developing innovative gut microbiota-targeted interventions for preventing or attenuating the global burden of kidney diseases.

## 1. Introduction

Our gastrointestinal tract contains trillions of microbes known as gut microbiota. The intestinal microbes have coevolved with humans for a mutually beneficial coexistence and are intimately involved in health and disease [1]. The shaping of the gut microbiome starts at birth, while the modification of its composition primarily relies on various early-life environmental factors [2]. Disturbances in the composition and function of the gut microbiota can impair intestinal permeability, microbial derived metabolites and immune responses, thereby yielding gut dysbiosis [3]. 

Pediatric renal diseases include a broad spectrum of disorders that influence mobility and mortality later in life. Since adult kidney diseases can originate in fetal life and childhood [4,5], the World Kidney Day 2016 alerted the public to the special attention necessary for pediatric renal diseases [6]. The latest clinical and experimental evidence has strengthened the association between numerous early-life factors and the development of kidney disease later in life [4,5]. Although the complete mechanisms are not conclusive yet, current evidence suggests that gut microbiota dysbiosis is emerging as a pathogenetic connection in the development of kidney diseases and associated conditions [7,8,9,10].

The bidirectional link between the kidney diseases and gut microbiota is named the kidney–gut axis [11]. In adults, prior research proposed some mechanisms linking gut microbiota dysbiosis to kidney disease, including inflammation, gut barrier dysfunction, alterations of microbiota composition, immune response, accumulation of trimethylamine N-oxide (TAMO), dysregulated short-chain fatty acids (SCFAs) and their receptors, and uremic toxins [7,8,9,10]. However, there is a paucity of data available about how the kidney–gut axis influences pediatric renal diseases and what the influence of gut dysbiosis is in modulating the pathological processes [8].

Over the years, gut microbiota-targeted therapies including prebiotics, probiotics, and postbiotics have gained attention due to their health benefits [12,13]. Considering that gut microbiota dysbiosis is involving in the pathogenesis of many kidney diseases, it seems logical to target gut microbiota for potential prevention and therapy of pediatric renal disease. The aim of our scoping review was to give insight on the relationship between gut microbiota and microbial metabolites in the pathophysiology of pediatric renal disease and on modulating the gut microbiota as a therapeutic approach to treatment.

## 2. The Kidney–Gut Axis

### 2.1. What Is Gut Microbiota?

The gut microbiota contains thousands of microbial species, and the trillions of microorganisms in the average human gut weigh about 1 kg. Gut microbes influence the absorption, metabolism, and storage of dietary nutrients, with profound influences in determining host physiology [1]. Additionally, the normal gut microbiota and its metabolites exert specific functions in the host, including maintenance of the gut mucosal barrier, immunomodulation, drug metabolism, blood pressure (BP) control, and protection against pathogens. 

Microbial diversity is evaluated based on two parameters: α- and β-diversity. The α-diversity is the variation within a single sample and is used to describe the compositional complexity, whereas β-diversity means the taxonomical differences between samples. Gut microbiota are composed of different bacterial species taxonomically classified by genus, family, order, and phyla [14]. In general, the healthy gut microbiota is predominantly constituted by the phyla *Firmicutes* and *Bacteroidetes*, followed by *Actinobacteria* and *Verrucomicrobia*. The compositional features of gut microbiota are distinct and differ among individuals, and although the mature microbiota is fairly resilient, it can be altered by both internal and external stimuli. Markers of microbial stability, such as diversity and richness, are often utilized as indicators of gut health due to their associations with many chronic diseases [1].

The gut microbiota differs between people and transforms during the human lifespan, under the influence of factors such as diet, environment, medication and diseases [15]. Diet is an important factor affecting the gut microbiota [15]. Dietary-derived microbial metabolites, notably SCFAs, TMAO, tryptophan derivatives, branched-chain amino acids and bile acids, have been strongly implicated in the pathogenesis of metabolic disorders. Dietary fiber is fermented by gut microbes, producing SCFAs [16]. SCFAs, and especially butyrate, participate in preserving intestinal immune homeostasis and exerting protective responses against oxidation and inflammation [16]. SCFAs can directly activate G-coupled receptors to regulate various physiological processes. These SCFA receptors are expressed in the kidney and have been reported to function as regulators of blood pressure (BP). Dietary L-carnitine and choline, which are found predominantly in animal-derived foods, can be metabolized to trimethylamine (TMA). TMA is metabolized to TMAO, which is subsequently converted to dimethylamine (DMA) [17]. High TMAO has been linked to increased cardiovascular risks and all-cause mortality [17]. 

Certain microbiota-derived tryptophan metabolites are uremic toxins, mostly coming from the indole and kynurenine pathways [18]. Many indole derivatives such as Indoxyl sulfate (IS) and indoleacetic acid (IAA) are acyl hydrocarbon receptor (AHR) ligands, having proinflammatory, prooxidant, procoagulant, and pro-apoptotic effects [19]. It is known that AHR can modulate the T helper 17 cell (TH17) and T regulatory cell axis, which participates in gut homeostasis and immune response [20]. Taken together, these findings make the point that the gut microbiota has a global influence on host physiological functions.

### 2.2. The Kidney–Gut Axis in CKD

Emerging human and experimental studies support that the kidney–gut axis participates in the pathogenesis of various renal diseases. One example is the well-known research in chronic kidney disease (CKD). There is a bidirectional relationship between gut microbiota and CKD [11]. CKD is able to affect gut microbiota in several ways. First, CKD is accompanied by increased intestinal permeability, namely leaky gut [21]. Bacteria and their cell-wall products such as lipopolysaccharide (LPS) can translocate from gut lumen into the bloodstream. The influx of bacteria and LPS into the circulation can activate innate immune cells through a toll-like receptor 4 (TLR4)-dependent mechanism, leading to kidney inflammation. Accordingly, a leaky gut leads to inflammation, malnutrition and accelerated CKD progression [21,22]. Second, uremic toxins are generated in CKD patients and adversely affect the growth of gut microbes. Lower richness (represented by α-diversity) of gut microbiota was reported in end-stage kidney disease (ESKD) patients than in control subjects [23]. Prior work indicated that CKD patients exhibited a lower abundance of beneficial microbes such as *Bifidobacterium* and *Lactobacillus* species [24]. Additionally, alterations of specific gut microbial taxa in patients with CKD were identified [23]. Furthermore, several CKD-related factors are involved in dysbiotic gut microbiota, including low fiber intake, malnutrition, metabolic acidosis, the use of antibiotics/medication, increased intestinal excretion of urea, accumulation of uremic toxins, and reduced gut motility [7,8,9,10,11].

**Figure 1:**
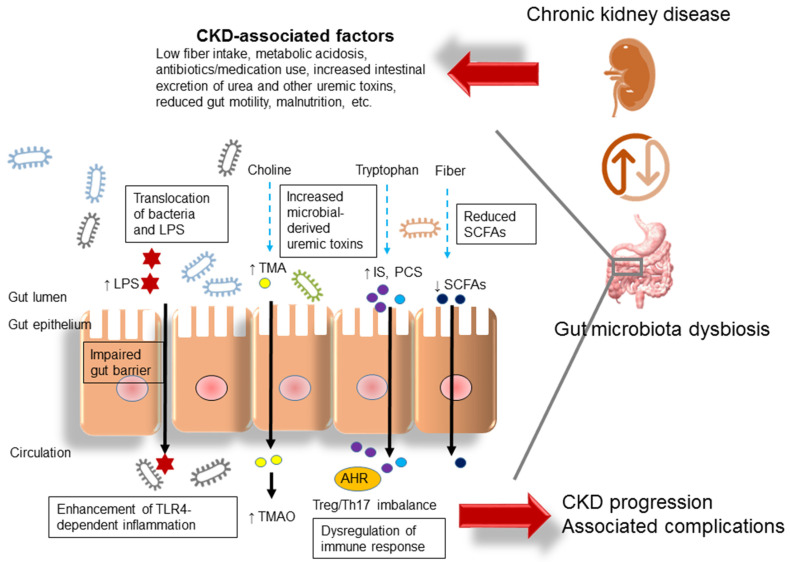
Illustration of proposed mechanisms behind the kidney–gut axis linking gut microbiota dysbiosis to chronic kidney disease (CKD). LPS = lipopolysaccharide; TLR4 = toll-like receptor 4; SCFA = short chain fatty acid; IS = indoxyl sulfate; PCS = p-cresyl sulfate; Th17 = T-helper 17 cell; Treg = regulatory T cell; AHR = aryl hydrocarbon receptor; TMAO = trimethylamine-N-oxide; TMA = trimethylamine.

On the other hand, gut microbiota-derived metabolites are involved in CKD progression and comorbidity. These microbial metabolite-related effects include: (1) microbial fermentation of dietary fibers to generate SCFAs that enhance SCFA receptors, increase energy expenditure and inhibit histone deacetylase (HDAC) activity; (2) microbial conversion of tryptophan-derived uremic toxins including IS and p-cresyl sulfate (PCS), which dysregulate Treg/TH17 balance through AHR activation and subsequently accelerate CKD progression; and (3) microbial conversion of choline to TMA, which is subsequently converted to TMAO, a well-known CVD risk factor. We summarized these interrelationships in Figure 1. 

### 2.3. The Kidney–Gut Axis in Other Kidney Diseases

An impaired gut–kidney axis with the dysbiotic gut microbiota is also implicated in other renal diseases, including acute kidney injury (AKI) [25], kidney transplantation (KT) [26], urinary stone disease [27], urinary tract infection (UTI) [28], and genitourinary cancers [29]. 

#### 2.3.1. Acute Kidney Injury

Compared to CKD, relatively few studies have examined the crosstalk between the kidney and gut in AKI. In an ischemia/reperfusion injury (IRI) mouse model, gut microbiota dysbiosis, characterized by a decrease in *Ruminococcaceae* and *Lactobacilli* and an increase in *Enterobacteriaceae*, was present on day 1 [30]. Another study showed that germ-free mice receiving stool from IRI mice had more severe kidney injury compared to controls. Accordingly, AKI may provoke gut dysbiosis in a short time period, and the shift in microbial composition can determine post-AKI severity [30].

#### 2.3.2. Kidney Transplantation

Unlike CKD, patients with KT have improvement in kidney function, placing them in earlier stages of CKD. However, previous work reported that KT can still cause substantial longitudinal alterations in microbial composition [31]. In view of the more complex clinical conditions of KT recipients, such as administration of immunosuppressive medications, posttransplant complications and frequent use of antibiotics, the imbalanced kidney–gut axis remains and deserves further evaluation. 

#### 2.3.3. Urinary Stone Disease

Although urinary stone disease is rare in the pediatric population, the relationship between gut microbiota and nephrolithiasis has been discovered in adults [27]. A meta-analysis study indicated that the gut microbiota in patients with stone formation is characterized by decreases in *Prevotella*, *Prevotellaceae*, and *Roseburia* and increases in *Enterobacteriaceae* and *Streptococcaceae* [27]. Given that urinary concentrations of oxalate, calcium, and uric acid have a key role in stone formation, increased net gastrointestinal absorption due to impaired microbial degradation of these stone compositions could influence their urinary excretion. Calcium oxalate stones are the most common type of kidney stone. Lack of oxalate-degrading microbes such as *Oxalobacter formigenes* has been linked to stone formation [32]. Under physiological conditions, one-third of uric acid is excreted through the intestines, as well suggesting the potential impact of gut microbiota in the pathogenesis of uric acid stones. Several observations have shown that the gut microbiota profile in patients with renal stones is distinct from that in control subjects, which further suggests that the gut microbiota has a crucial contribution in stone formation [33].

#### 2.3.4. Urinary Tract Infections

Uropathogenic Escherichia coli (UPECs) are the main causative agent of UTIs. These UPEC strains are abundant in the gut of patients with UTIs and are thus considered to originate from the gut [34]. This notion was supported by a previous study demonstrating that increased abundance of E. coli in the gut was related to future development of E. coli-induced UTI, and E. coli strains in the urine bore a close resemblance to those in the gut from the same subject [35]. 

#### 2.3.5. Genitourinary Cancers

Moreover, the potential influence of the gut microbiome on the pathobiology of genitourinary cancers has been extensively studied in adults [29], although these diseases in children are uncommon. As many carcinogens are produced in the gut and filtered by the urinary tract prior to excretion, gut bacteria might not only participate in the development of genitourinary cancers but also act as therapeutic targets for the prevention of these cancers. 

## 3. The Impact of Gut Microbiota in Pediatric Renal Disease

It is now widely appreciated that the gut microbiota is involved in human health and disease. Although the kidney–gut interaction clearly has a crucial role in the onset and progression of several adult kidney diseases, the relationship between gut microbiota and kidney health has yet to be completely elucidated in the pediatric population.

Table 1 lists a summary of the alterations in gut microbiota compositions and metabolites in various pediatric renal diseases [36,37,38,39,40,41,42,43,44,45], based on available information extracted from the literature. The main findings regarding gut microbiota profiles mostly focused on three types of gut dysbiosis: alterations in keystone taxa, diversity loss and changes in gut microbiota-derived metabolites.

One study recruiting 86 children and adolescents with CKD demonstrated those with CKD stage 2–3 had lower urinary concentrations of TMAO and DMA than those with CKD stage 1 [36]. Additionally, the abundance of genus *Prevotella* was negatively correlated with BP abnormalities determined using 24 h ambulatory blood pressure monitoring (ABPM). In another group of children and adolescents with CKD stage 1–4 (*n* = 78), BP abnormalities were correlated with increased plasma concentrations of butyric acid and propionic acid [37]. In a 1-year follow-up study, there was a positive correlation between high index plasma butyrate and worsened BP. At the follow-up, plasma butyrate concentration was reduced by 2.12 and 4.41 μM in the stable and worsened BP groups, respectively [39]. These findings suggest that index plasma butyrate concentration appears to correlate to adverse cardiovascular outcomes [39]. In addition, CKD children with non-glomerulonephritis had a higher abundance of phylum *Verrucomicrobia*, genus *Akkermansia*, and species *Bifidobacterium bifidum* compared with those with glomerulonephritis [37]. 

Consistent with prior research in CKD adults [46], plasma TMAO level has a negative correlation with renal function in youth, as one study showed children with CKD stage 2–4 had greater TMAO and TMA levels compared to those with CKD stage 1 [38]. This study, recruiting 115 children and adolescents with CKD stage 1–4, demonstrated that those with hypertension had a low proportion of phylum *Cyanobacteria*, genera *Ruminococcus*, *Subdoligranulum*, *Akkermansia*, and *Faecalibacterium*. Additionally, CKD children with BP abnormalities displayed a low proportion of TMA-producing microbes [47], such as *Gemella*, *Providencia*, and *Peptosreptoccocus*. In another study, of 38 children and adolescents with advanced CKD stage 3–5, it was shown that tryptophan-derived metabolites were increased in a stage-dependent manner [40].

Exposure to the uremic milieu in the course of end-stage kidney disease (ESKD) was evaluated in 16 children and adolescents who underwent peritoneal dialysis (PD, *n* = 8) or hemodialysis (HD, *n* = 8) [41]. Children undergoing PD or HD had increased plasma concentrations of uremic toxins originating from gut microbial metabolism, IS, and PCS [41]. PD led to a decline in α-diversity and an abundance of bacteria of the family *Enterobacteriaceae*. In addition, ESKD children undergoing HD had an increased abundance of phylum *Bacteroidetes*, while those undergoing PD displayed an increased proportion of phyla *Firmicutes* and *Actinobacteria*. There were only two studies evaluating gut microbiota in children receiving KT [40,41]. Although these children had improvements in kidney function, KT still disrupted the gut barrier, reduced α-diversity and impaired the microbial metabolite balance [40,41].

To date, several pediatric studies have been conducted to evaluate the impact of gut microbiota in idiopathic nephrotic syndrome (INS). One report showed that relapsing INS children had a low proportion of butyrate-producing microbes and fecal butyric acid concentration [42]. These microbes belong to *Clostridium* clusters IV and XIVa, including *Clostridium orbiscindens*, *Eubacterium hallii*, *Roseburia intestinalis*, *E. ramulus*, *E. rectale*, *E. ventriosum*, *Faecalibacterium prausnitzii*, *Roseburia intestinalis*, *Eubacterium* spp., and *Butyrivibrio* spp. 

A prior study demonstrated that 20 children with INS had increases in the abundance of genera *Romboutsia*, *Stomatobaculum*, and *Cloacibacillus* after a 4 weeks of oral prednisone therapy [43]. In 20 children with INS, probiotic treatment was shown to protect against relapse and was accompanied by increases in butyrate-producing microbes and blood regulatory Treg cell counts [44]. As the balance between Treg and TH17 is involved in renal inflammation, probiotic treatment could have beneficial effects impacting the kidney–gut axis via immune regulation. Moreover, the immunological impact of a gluten-free and dairy-free (GF/DF) diet on gut microbiota and the Treg/TH17 balance was assessed in children with steroid-resistant nephrotic syndrome (SRNS) [45]. In 16 children with SRNS, a 4-week GF/DF dietary intervention altered gut microbiota composition, characterized by increases in *Lachnospira*, *Bacteroides* and *Faecalibacterium* [45]. Additionally, a GF/DF diet exhibited anti-inflammatory effects with a 4-fold increase in the Treg/TH17 ratio.

Not only have microbial communities in the gut been implicated in pediatric renal diseases, but the urine microbiome has also been associated with urinary tract infections (UTI), vesicoureteral reflux, and neurogenic bladder [48,49]. Nevertheless, no information so far exists regarding the impact of gut microbiota on these diseases.

## 4. Gut Microbiota-Targeted Therapy

**Figure 2:**
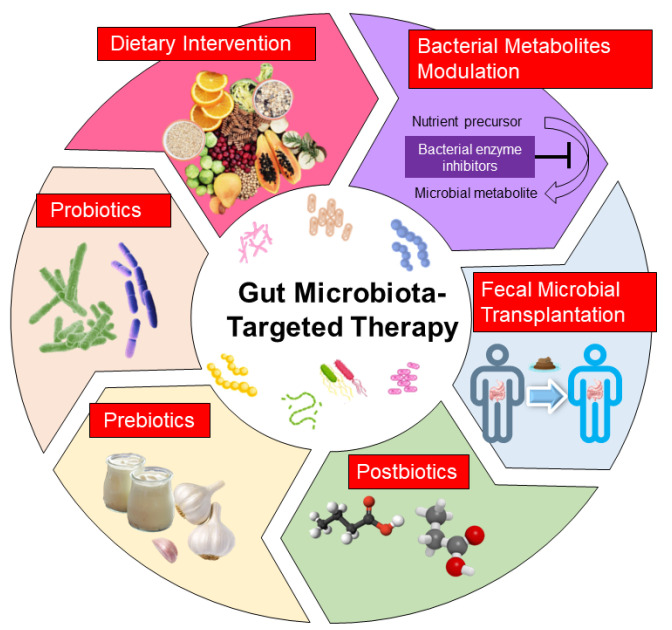
Illustration of current potential gut microbiota-targeted therapies to improve pediatric renal disease.

In view of the high correlation between gut microbiota and kidney disease, potential therapies designed to modulate gut microbiota and microbial metabolites could be promising strategies for the prevention of kidney disease. In clinical practice, several gut microbiota-targeted therapies have been applied in kidney diseases, including dietary intervention, probiotics and prebiotics. Additionally, postbiotics, fecal microbiota transplantation (FMT) and bacterial metabolite modulation have been examined in experimental kidney diseases. Each therapy is illustrated in Figure 2 and discussed in turn.

### 4.1. Dietry Interventions

Dietary interventions work as the first-line treatment for CKD, due to their role in improving CKD outcomes. A plant-dominant, low-protein diet was reported to alter the gut microbiome, which could modulate uremic toxin generation and slow CKD and CVD progression [50]. A high-fiber diet may prevent the progression of CKD in people with CKD [51] and was related to restoration of gut microbiota and increased the abundance of SCFA-producing bacteria [52]. Dietary sulfur-containing amino acid intake has also been reported to ameliorate CKD progression and its associated complications in adult patients and disease models [53,54]. In accordance with the above-mentioned studies, dietary interventions indeed impact the gut microbiota and show benefits with regard to CKD progression. However, whether these dietary interventions are beneficial in pediatric kidney disease has not yet been adequately addressed. So far, only one study reported that a gluten-free and dairy-free diet influences gut microbiota and the Treg/TH17 balance in children with SRNS [45].

### 4.2. Probiotics

Probiotics are defined as live bacteria that have health benefits when administered [55]. The major probiotics include one or more strains arising out of the genera *Bifidobacterium*, *Lactobacillus* and *Streptococcus* [55]. Certain probiotic microorganisms may have beneficial effects on adult CKD [56,57], while evidence regarding their role on pediatric CKD is quite rare. 

One example of a probiotic is *Clostridium butyricum*, a butyrate-producing microbe [58]. Supplementation with *Clostridium butyricum* during remission could reduce the frequency of relapse and the use of immunosuppressive drugs in children with INS [44]. The beneficial actions of *Clostridium butyricum* were related to increased Treg cells and butyrate-producing bacteria. 

In addition to CKD, certain probiotics such as *Lactobacillus salivarius* and *Bifidobacterium bifidum* have shown their beneficial effects in attenuating AKI in animal models [59,60], while their role in pediatric AKI remains unknown.

### 4.3. Prebiotics

Prebiotics are selectively fermented ingredients that have beneficial effects by stimulating the growth or activity of gut microbes [61]. As reviewed elsewhere [62], evidence showed that prebiotic inulin, fructooligosaccharides, resistant starch, and soluble fiber could reduce uremic toxins and promote growth of beneficial microbes in adult patients with CKD. Additionally, some prebiotic foods, such as resveratrol and garlic oil, have shown benefits in protection against pediatric CKD and associated complications in animal models [63,64]. Although probiotic treatment exhibits beneficial effects in CKD adults and animal models, much remains to be elucidated about their roles in pediatric CKD.

Unlike CKD, the impact of prebiotics on AKI is still unclear. So far, only an ongoing double-blind, randomized controlled trial that evaluates prebiotics and probiotics in patients with septic AKI might give a more exact answer (https://clinicaltrials.gov/ct2/show/NCT03877081, accessed on 10 January 2023). 

### 4.4. Postbiotics

The utilization of substances produced by or derived from bacteria, namely postbiotics, has revealed health benefits [65]. Postbiotics can include various constituents such as metabolites, cell fractions, extracellular vesicles, bacterial lysates, and extracellular polysaccharides [65]. Short-chain fatty acids (SCFAs) such as acetate, propionate and butyrate are well-known postbiotic metabolites. 

Postbiotics are supposed to aid in preservation of the gut barrier, reduction of inflammation, and regulation of blood sugar, and help with several conditions that range from digestive illnesses to various chronic diseases [65]. As a postbiotic, the protective effect of SCFA supplementation against AKI [66] and CKD [67] has been examined in animal models. Experimental evidence indicating that butyrate or propionate supplementation prevents hypertension is related to increased renal expression of SCFA receptor G protein-coupled receptor 41 [67,68]. Nevertheless, currently, no information exists regarding the use of postbiotics in pediatric renal diseases. 

### 4.5. Fecal Microbiota Transplantation

FMT is able to rebuild the microbiota diversity and composition by transferring healthy gut microbiota into recipients. To date, FMT has been widely studied in various microbiome-associated clinical disorders, not only in adults [69] but also in the pediatric population [70]. However, evidence regarding FMT for the treatment of human kidney disease remains very limited.

One case report showed that FMT ameliorated nephrotic syndrome and improved kidney function in a patient with membranous nephropathy [71]. Another report demonstrated that two immunoglobulin A (IgA) nephropathy patients undergoing FMT displayed partial remission, characterized by a decrease in proteinuria and stable kidney function [72]. These examples of preliminary evidence regarding the safety and efficacy of FMT for treating adult kidney disease may open a new avenue for it to be applied in the pediatric population.

### 4.6. Bacterial Metabolite Modulation

AST-120 is an oral sorbent that can adsorb small organic molecules accumulating in the gut of patients with CKD [73]. In human and experimental CKD, AST-120 could reduce uremic toxins and display cardiovascular benefits [74,75,76]. However, its impact on CKD progression remains inconclusive [77]. Further research is needed to understand the potential benefits of absorption of toxic microbial metabolites to delay CKD progression in children. 

Recently, researchers turned their attention to the modulation of TMAO [78]. As TMA is converted to TMAO, inhibition of intestinal TMA production by microbial choline TMA lyase inhibitors, such as 3, 3-dimethyl-1-butanol (DMB) or iodomethylcholine (IMC), has been used to inhibit TMAO production in animal models [78,79,80]. Our recent work demonstrated that IMC treatment reduced TMAO, altered gut microbiota composition, and averted offspring hypertension in an adenine-induced CKD model [81]. These findings theoretically revealed that targeted TMAO reduction might have therapeutic potential for the prevention of pediatric CKD, even if these results require further clinical translation.

Although current evidence suggests that antibiotic treatment decreases the diversity of gut microbiota, resulting in gut dysbiosis [82], a previous study indicated that vancomycin could remove gut microbiota-derived uremic toxins, IS and PCS in patients with ESKD [83]. Considering that various antibiotics may differentially alter gut microbiota composition and its derived metabolites, it will be interesting to see which antibiotic can be utilized as a therapeutic intervention for bacterial metabolite modulation to improve pediatric CKD. 

## 5. Conclusions and Perspectives

Growing evidence supporting the connection between microbial dysbiosis and pediatric renal disease is intriguing but incomplete. Our literature review summarizes the currently reported gut microbiome and its derived metabolites, which are manifested in children with kidney disease. The reported pediatric cases with kidney disease are dominated by CKD and INS. The major findings regarding gut microbiota profiles mostly focused on the loss of microbiota diversity, alterations in keystone taxa (e.g., butyrate-producing bacteria) and changes in gut microbiota-derived metabolites (e.g., SCFAs and TMAO). These microbiome profiles are more or less the same as those of kidney disease in adults. It should be acknowledged that the number of pediatric cases with kidney disease is very small in relation to the thousands of adults being reported. Specifically, there have been reported BP abnormalities associated with alterations of gut microbiota in children in the early stage of CKD [36,37,38], which reinforces the importance of gut microbiota in this vulnerable group. To date, no gut microbiota research related to AKI, UTI, urinary stone disease, and genitourinary cancers in the pediatric population have been reported. 

In the future, we recommend bridging the gap between child and adult research focused on kidney disease. The fraction of articles focused on the pediatric population is less than 10%. Notably, most pediatric studies have small sample sizes, which can reduce the power of a study and render it meaningless. Noting that a considerable body of evidence has been developed and indicates a strong connection between gut microbiota and kidney diseases in adult patients, there remains a lack of such information in the pediatric field. Hence, carrying out large multicenter pediatric kidney studies is required to confirm the real link between the microbial community and kidney disease in the pediatric population. 

Gut microbiota modulation by means of dietary interventions, probiotics, prebiotics, postbiotics, and FMT may perhaps maintain kidney health. However, several probiotics and prebiotics utilized in adults have not yet been studied in children, particularly in CKD. Considering that the early-life gut microbiome affects kidney development and programs kidney disease in later life [5], more attention should be paid to evaluate the value of gut microbiota-targeted therapies in childhood rather than later in life. Our recommendations are that microbiota studies be conducted to identify the gut microbiota community and metabolites at different disease stages, especially at the early stage, and that gut microbiota-targeted therapy be provided for children with CKD in the early stages. These directions may provide significant clues to the etiology of disease, molecular mechanisms and potential treatment for pediatric renal disease.

In summary, we highlighted a pathogenetic link between gut microbiota dysbiosis and the development of pediatric renal disease. Significant strides made in our understanding of the gut microbiota involved in pediatric renal disease and its targeted interventions may open new avenues for averting various kidney diseases not only in childhood, but even later in life.

## Figures and Tables

**Table 1:** Summary of Pediatric Renal Disease Studies on Gut Microbiota.

Pediatric Renal Disease	Study Population	Main Significant Findings	References
CKD	86 children and adolescents with CKD stage 1–3	↓Urinary concentrations of TMAO and DMA in CKD stage 2–3; ↓Genus *Prevotella* in CKD children with hypertension	Hsu et al., 2018 [36]
CKD	78 children and adolescents with CKD stage 1–4	↑Phylum *Verrucomicrobia*, genus *Akkermansia*, and ↓species *Bifidobacterium bifidum* in CKD children with non-glomerulonephritis; ↑Plasma levels of butyric acid and propionic acid in CKD children with hypertension	Hsu et al., 2019 [37]
CKD	115 children and adolescents with CKD stage 1–4	↑Plasma concentrations of DMA, TMA, and TMAO in children with CKD stage 2–4; ↓Phylum *Cyanobacteria*, genera *Subdoligranulum*, *Ruminococcus*, *Faecalibacterium*, and *Akkermansia* in CKD children with hypertension	Hsu et al., 2020 [38]
CKD	105 children with CKD stage 1–4	The index of higher plasma butyrate was positively correlated with worsened blood pressure at 1-year follow-up.	Lu et al., 2021 [39]
CKD/KT	38 children and adolescents with CKD stage 3–5 (12 CKD stage 3–4, 11 HD, 15 KT); 10 controls	Gut barrier dysfunction and microbial metabolite imbalance. Plasma metabolite analysis showed a stage-dependent increase in tryptophan metabolites.	Holle et al., 2022 [40]
ESKD/KT	26 children and adolescents with ESKD (8 HD, 8 PD, 10 KT); 13 controls	↓α-diversity in PD and KT; ↑Phylum *Bacteroidetes* in HD; ↑Plasma levels of indoxyl sulfate and p-cresyl sulfate in HD and PD; ↓Phyla *Firmicutes* and *Actinobacteria* in PD;↑family *Enterobacteriaceae* in PD	Crespo-Salgado et al., 2016 [41]
INS	12 children and adolescents with INS; 11 controls	↓Fecal butyric acid level; ↓Butyrate-producing bacteria belonging to *Clostridium clusters IV* and *XIVa*	Tsuji et al., 2018 [42]
INS	20 children and adolescents with INS	↑Genera *Stomatobaculum Romboutsia*, and *Cloacibacillus* after 4-week initial therapy	Kang et al., 2019 [43]
INS	20 children and adolescents with INS	↓Butyrate-producing bacteria	Yamaguchi et al., 2021 [44]
SRNS	16 children and adolescents with SRNS	↑*Lachnospira*, *Bacteroides* and *Faecalibacterium* after a 4-week dietary intervention	Pérez-Sáez et al., 2021 [45]

Studies tabulated according to types of pediatric renal disease and year of publication. CKD = chronic kidney disease; KT = kidney transplantation; INS = idiopathic nephrotic syndrome; SRNS = steroid-resistant nephrotic syndrome; HD = hemodialysis; PD = peritoneal dialysis; DMA = dimethylamine; TMAO = trimethylamine N-oxide; TMA = trimethylamine; ↑ = increased; ↓ = decreased.

## Data Availability

Data are contained within the article.
